# Prognostic factors and treatment effects for hepatocellular carcinoma in Child C cirrhosis

**DOI:** 10.1038/sj.bjc.6604282

**Published:** 2008-03-18

**Authors:** K Nouso, Y M Ito, K Kuwaki, Y Kobayashi, S Nakamura, Y Ohashi, K Yamamoto

**Affiliations:** 1 Department of Internal Medicine, Hiroshima City Hospital, Hiroshima, Japan; 2 Department of Biostatistics, School of Public Health, The University of Tokyo, Tokyo, Japan; 3 Department of Medicine and Medical Sciences, Okayama University Graduate School of Medicine, Dentistry, and Pharmaceutical Sciences, Okayama, Japan

**Keywords:** decompensated cirrhosis, prognostic factors, hepatocellular carcinoma, therapy

## Abstract

The aim of this study is to elucidate the prognostic factors and the treatment effect on survival in hepatocellular carcinoma (HCC) patients with Child C cirrhosis. Out of 3330 newly discovered HCC patients, 157 consecutive HCC individuals with Child C cirrhosis were enrolled. The prognostic factors were examined by Cox proportional hazards regression analysis and their survival was compared by propensity score-matched analysis. Multivariate analysis revealed that high serum bilirubin (>3 mg dl^−1^), the presence of uncontrollable ascites, and a high platelet count (>8 × 10^4^ mm^−3^), so-called background liver factors, as well as multiple tumours, large tumours (>3 cm), high alpha-fetoprotein (>400 ng ml^−1^), and the presence of portal vein thrombus, so-called tumour factors, were factors of poor prognosis. While transcatheter arterial chemoembolisation (TACE) was a factor of good prognosis (relative risk=0.50, 95%CI=0.27–0.89, *P*=0.019), local ablation therapy and transcatheter arterial chemoinfusion (TAI) were not significant prognostic factors. The survival of patients who received TACE was superior to matched patients without active treatment (*P*=0.009); however, we did not observe survival benefit after local ablation therapy or TAI. These results suggested that tumour factors as well as background liver factors are prognostic factors of HCC even in patients with Child C cirrhosis, and selective use of TACE in these patients provides survival benefit.

Hepatocellular carcinoma (HCC) is the fifth cause of death by cancer worldwide (Parkin, 2001). Many symptomatic HCCs are diagnosed in advanced stage and cannot be treated, so the prognosis is generally poor (Wong *et al*, 2000; Bruix and Llovet, 2002). By the accumulation of knowledge of the risk factors and the prevalence of HCC surveillance, the proportion of HCC diagnosed in early stage and that can be treated by local ablation therapies or surgery has increased (Bolondi, 2003; Adams *et al*, 2004; Trevisani *et al*, 2004).

In spite of the early detection of HCC, many patients die of complications of severe cirrhosis without any active treatment (Ikai *et al*, 2007). According to the algorithms of the treatment of HCC recommended by groups in Europe and Japan (Bruix *et al*, 2001; Llovet, 2005; Makuuchi and Kokudo, 2006), HCC patient with Child C cirrhosis (Pugh *et al*, 1973) is a candidate for liver transplantation or best supportive care (BSC). However, even though HCC meets the Milan criteria, many patients do not receive liver transplantation because of the shortage of donors or advanced age (United Network for Organ Sharing, 2006). Although there is regional variability, patients diagnosed with HCC in the background of hepatitis C virus infection are usually elderly (Parkin, 2001). In Japan, the mean age of patients is 66.6 years old (Ikai *et al*, 2007); therefore, a large number of the patients are outside the liver transplantation criteria and receive only BSC.

Local ablation therapies and transcatheter arterial chemoembolisation (TACE) are known to be useful for HCC treatment with preserved liver function (Llovet *et al*, 2003). Nevertheless, these therapies for patients with Child C cirrhosis are not recommended because of reported severe adverse events. However, there is lack of knowledge on prognostic factors on the effectiveness of active treatment, except liver transplantation, in HCC patients with Child C cirrhosis (Llovet, 2005). Therefore, it is very important to identify factors influencing outcomes of cirrhotic patients affected by HCC who cannot be treated by liver transplantation.

In this study, we retrospectively examined the clinical course of HCC patients with Child C cirrhosis and analysed their prognostic factors.

## PATIENTS AND METHODS

### Patients

Between January 1996 and September 2006 among 3330 consecutive newly diagnosed HCC patients who were admitted to our department and affiliated hospitals, 186 individuals had Child C cirrhosis. Five patients were excluded because of a lack of clinical data, 24 were excluded because they underwent liver transplantation, and the remaining patients (*n*=157) were enrolled in this study. Informed consent was obtained from all patients for use of their clinical data. The study protocol conformed to the ethical guidelines of the World Medical Association Declaration of Helsinki, and was approved by the ethical committees of the institutes.

### Diagnosis

One hundred and forty-one patients were diagnosed as having HCC by imaging modalities, such as angiography, computed tomography (CT), and magnetic resonance imaging (MRI). Diagnostic criteria for HCC via imaging was based on previous reports of hyperattenuation at the arterial phase, hypoattenuation at the portal phase in dynamic CT (section thickness=5–8 mm) or MRI, and tumour stain on angiography (Honda *et al*, 1993). The remaining 16 patients with hepatic masses who did not satisfy the above criteria underwent ultrasound (US)-guided fine-needle biopsy with histologically confirmed HCC.

### Treatments

As there is no clear evidence that active treatment of HCC improves the survival of patients with Child C cirrhosis, all the patients were informed that the potential treatment benefits were unknown and the risks of the treatments were higher than in patients with Child A/B cirrhosis. We conducted local ablation therapy, TACE, or transcatheter arterial chemoinfusion (TAI) only when patients consented to undergo these interventions. Percutaneous ethanol injection therapy, radiofrequency ablation, and microwave coagulation therapy were performed in 18, 4, and 1 patients, respectively. All HCCs treated by local ablation therapy were less than 3 cm in diameter and less than three tumours, except three single large HCC with diameters between 3.0 and 3.5 cm. There are two major differences of our treatment algorithm for patients with Child C cirrhosis from that for patients with Child A/B cirrhosis. Surgical resection was not chosen and indication for local ablation therapy was stricter. When a tumour protruded from the liver surface, attached to the adjacent organs such as gall bladder, or a tumour was not confirmed well by ultrasonography, local ablation therapies were not performed. The therapies were also avoided when uncontrollable ascites was present. We included transcatheter arterial embolisation (TAE) in the group undergoing TACE, because the number of patients who received TAE was small and no clear difference in the treatment effect was reported in previous studies (Camma *et al*, 2002). Transcatheter arterial chemoembolisation was not chosen principally in cases of severe portal vein tumour thrombus (PVTT). Transcatheter arterial chemoembolisation and TAI were performed supraselectively in the most peripheral accessible feeding artery to avoid irreversible liver failure. When the treatment might cause immediate irreversible liver failure or severe complications, we did not perform any active treatments irrespective of the patients’ wishes, except emergency TACE for HCC rupture.

### Follow-up

Biochemical liver function tests and US, dynamic CT, or MRI were performed at least every 3 months after the treatment. Re-treatment was performed depending on patients’ conditions, tumour stage and background liver function, according to the same clinical indications as for the first intervention.

### Statistical analysis

The Kruskal–Wallis test was used to compare the continuous data and the *χ*^2^ test was used to compare categorical data. Cox proportional hazards regression analysis was used to analyse the prognostic factors. Factors exhibiting significant values in univariate analysis and effect of the treatments were further analysed by multivariate analysis. The propensity score of choosing each treatment was calculated, followed by matching each treatment group and BSC group according to a greedy matching technique (Parsons, 2001). For calculation of the propensity score, following variables (cutoffs) were used: total bilirubin (<2, 2–3, >3, mg per dl), tumour size (<20, 20–30, >30, mm), tumour number (1, >1), PVTT (absent, present). The survival of matched patients was compared by the Kaplan–Meyer method and the differences were evaluated by the log-lank test. SAS (version 9.1.3) and JMP (version 5.0.1) software packages (SAS Institute, Cary, NC, USA) were used for analyses, and *P*<0.05 was considered significant. Bonferroni correction was used for multiple comparisons of propensity score-matched analyses and *P*<0.05/3 was considered significant.

## RESULTS

### Clinical characteristic of the patients

Twenty-three patients (14.7%) were treated by local ablation therapy, 27 (17.2%) by TACE, and 19 (12.1%) by TAI. The remaining patients (*n*=88, 56.1%) did not receive any of these treatments (BSC group). One- and 3-year survival of patients was 42.6 and 14.0%, respectively.

The characteristics of all patients are reported in Table 1. There was no difference in sex and age among these groups. The positive rate of hepatitis C virus antibody was low (55.7%) in the BSC group. Background liver function (Child-Pugh score) was worse and the tumours were more advanced (tumour size, tumour number, alpha-fetoprotein (AFP), and PVTT) in the BSC group than in other groups. Eighteen patients could be re-treated by local ablation therapies, TAE, or TAI.

### Risk factors for survival of HCC patients with Child C cirrhosis

Among 17 parameters and treatment modalities, high bilirubin (>3 mg dl^−1^), the presence of uncontrollable ascites, and a high platelet count (>8 × 10^4^ mm^−3^), so-called background liver factors, as well as multiple tumour number, large tumour (>3 cm), high AFP (>400 ng ml^−1^), and the presence of PVTT, so-called tumour factors, were significant risk factors for death in univariate analysis in Table 2. Conversely, TACE and local ablation therapy were associated with better survival. As shown in Table 3, multivariate analysis revealed that all background factors and tumour factors that were significant in univariate analysis were also significant risk factors. Regarding therapies, only TACE was a significant negative risk factor for death by multivariate analysis.

### Survival of patients in different therapeutic groups

**Figure 1:**
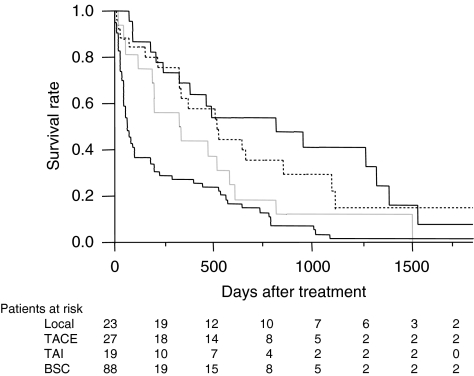
Survival of HCC patients with Child C cirrhosis. One-year (3-year) survival of patients receiving local ablation therapy (solid line), TACE (dotted line), transcatheter chemoinfusion (grey line), and BSC (thin line) was 69.1 (41.3), 62.5 (29.8), 43.9 (12.6), and 27.7% (3.8%), respectively (*P*<0.001).

**Figure 2:**
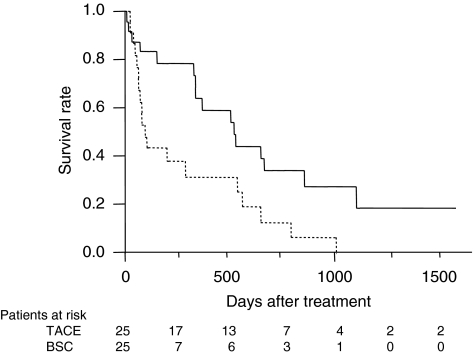
Survival of propensity score-matched patients treated by TACE or BSC. The survival of the TACE group (solid line) was significantly better than that of the BSC group (dotted line, *P*=0.009).

**Figure 3:**
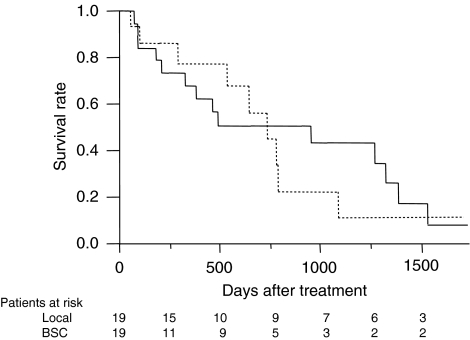
Survival of propensity score-matched patients treated by local ablation therapy (Local) or BSC. No differences were observed between the Local group (solid line) and BSC group (dotted line, *P*=0.782).

**Figure 4:**
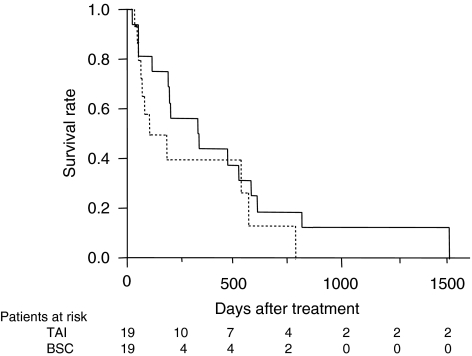
Survival of propensity score-matched patients treated by TAI or BSC. No differences were observed between TAI group (solid line) and BSC group (dotted line, *P*=0.237).

One-year (3-year) survival of patients receiving local ablation therapy, TACE, TAI, and BSC was 69.1 (41.3), 62.5 (29.8), 43.9 (12.6), and 27.7% (3.8%), respectively (*P*<0.001, Figure 1). To estimate the effect of treatments, the clinical background of the patients in each group was adjusted by propensity scores, and the survival of treated groups was compared with the BSC group. Numbers of the score-matched pairs were 25, 19, and 19 for TACE *vs* BSC group, local ablation therapy *vs* BSC group, and TAI *vs* BSC group, respectively. One patient in the BSC group for the comparison with TACE group had a main portal vein thrombus. While survival in the TACE group was significantly better than in the BSC group (*P*=0.009, Figure 2), no differences in survival were observed between the local ablation group and BSC group (*P*=0.782, Figure 3), and between TAI group and BSC group (*P*=0.237, Figure 4).

## DISCUSSION

The prognosis of HCC patients with Child C cirrhosis is well known to be poor; however, there is little information about the prognostic factors among patients and the effect of active treatment, except liver transplantation (Llovet, 2005). In this study, we clearly demonstrated that high total bilirubin (>3 mg dl^−1^) and the presence of uncontrollable ascites, categorised as background liver factors, were independent factors for poor prognosis in HCC patients with Child C cirrhosis. In addition, tumour factors such as tumour size (>3 cm), tumour number (multiple), AFP (>400 ng ml^−1^), and PVTT were also independent prognostic factors. These factors were quite similar to the reported prognostic factors for HCC, which include chronic hepatitis and Child A or B cirrhosis (Sala *et al*, 2005). High platelet count also correlated with poor prognosis. Platelet is known to decrease according to the advancement of cirrhosis. Therefore, it is possible that the liver in patients with high platelet count is not cirrhotic and the liver function is disturbed by very advanced HCC, which can be a reason of poor prognosis. This explanation was strengthened by a result in this study. The size of HCC in patients with high platelet count was significantly larger than that in patients with low platelet count (data not shown).

The contribution of tumour factors to HCC patients with Child C cirrhosis indicated that HCC treatment might prolong survival even though the patients suffered from Child C cirrhosis. The results of multivariate analysis of the treatments and of propensity score-matched survival curves support this hypothesis. The relative risk for patients undergoing TACE was 0.50 and survival of this group was better than that of BSC (*P*=0.009). Only two patients died within a month and had been treated by TACE because of HCC rupture (data not shown). Although TACE might be an eligible method for the treatment of HCC with Child C cirrhosis, the results do not indicate that TACE is effective in all Child C patients. Transcatheter arterial chemoembolisation was selected for patients without severe portal vein thrombus and with relatively good liver function in this analysed population. The medians of bilirubin and prothrombin time in TACE group were 1.9 mg dl^−1^ and 64.2%, respectively.

While TACE showed a beneficial effect, local ablation therapy did not prolong survival. One possible reason is that the deterioration of liver function to death is much faster than tumour progression. The reported 1-year local recurrence rate of HCC in patients treated by local ablation therapy was low (2–18%) (Lin *et al*, 2005; Tateishi *et al*, 2006; Kim *et al*, 2006b), whereas the 1-year survival rate of Child C patients treated by local ablation therapy was 69.1%, indicating that many patients died without recurrence of HCC. Although no effect of local ablation therapy was observed, therapy including RFA could be used for decompensated liver cirrhosis (Kim *et al*, 2006a) and it is possible that it might be beneficial in special circumstances, such as when minute growth of the tumour immediately results in the occlusion of major critical vessels.

Several studies have addressed the characters of HCC with decompensated cirrhosis (Nagasue *et al*, 1999; Ueno *et al*, 2002; Toyoda *et al*, 2005). Ueno *et al* (2002) reported that high albumin, lack of oesophageal varices, small tumour, single tumour, and low AFP were survival factors for HCC patients with decompensated liver cirrhosis. The study included many Child B cirrhosis patients (over 85%); however, the results were quite similar to our data with Child C cirrhosis. Toyoda *et al* (2005) reported risk factors for HCC patients with Child C cirrhosis and beneficial effect of treatment; however, the study included old cases and details of the treatment were not described. Regarding therapies, BSC was recommended for the treatment of HCC with decompensated cirrhosis, except in transplantation-eligible cases, by the algorithms of HCC treatment demonstrated by groups in Europe and Japan (Bruix *et al*, 2001; Llovet, 2005; Makuuchi and Kokudo, 2006), while there are several reports indicating the usefulness or safety of operation, RFA, and TACE for patients with decompensated liver cirrhosis (Nagasue *et al*, 1999; Ueno *et al*, 2002; Kim *et al*, 2006a). Prospective randomised study is the best method to know the benefit of these therapies for HCC patients with Child C cirrhosis; however, it is ethically difficult now because no clear evidence of the beneficial effect of active treatments was reported and most of the guidelines for the treatment of HCC did not recommend these therapies except transplantation. Our study was a retrospective cohort study, the patient groups were heterogenous and the number of patients in each arm was quite limited; however, we clearly indicates the possibility of adopting TACE for the treatment of HCC in patients with Child C cirrhosis by both multivariate Cox proportional hazard model and propensity score-matched analyses.

Recently, improvement of liver function in patients with decompensated liver cirrhosis by anti-hepatitis virus therapy such as lamivudine or adefovir dipivoxil was reported (Hiraoka *et al*, 2005; Takamura *et al*, 2007). Adoption of these anti-viral therapies can reduce patient mortality from liver failure so that the treatment effect of local ablation therapy may improve, resulting in increased candidates for active treatment of HCC with Child C cirrhosis.

In this study, we demonstrated that tumour factors as well as background liver factors were risk factors even in HCC patients with Child C cirrhosis, and that TACE can be effective in a very selected group of patients. A randomised controlled study is needed to expand the eligible criteria for active treatment.

## Figures and Tables

**Table 1:** Clinical background of 157 patients

	**TACE**	**TAI**	**Local**	**BSC**	***P*-value**
Patient number	27 (17.2%)	19 (12.1%)	23 (14.7%)	88 (56.1%)	
Sex (male)	21 (77.8%)	15 (79.0%)	16 (69.6%)	64 (72.7%)	0.085
Age (years)	66 (55–70)	65 (60–68)	59 (52–64)	62 (56–70)	0.275
HCVAb (positive)	24 (88.9%)	15 (79.0%)	18 (78.3%)	49 (55.7%)	0.003
HBsAg (positive)	2 (7.4%)	2 (10.5%)	4 (17.4%)	25 (28.4%)	0.061
Total bilirubin (mg per dl)	1.9 (1.2–2.5)	2.4 (2.1–3.1)	2.8 (2.1–3.4)	3.5 (2.2–4.8)	<0.001
Albumin (g per dl)	2.6 (2.5–2.7)	2.5 (2.3–2.9)	2.7 (2.5–3.0)	2.6 (2.2–2.9)	0.213
AST (IU per l)	61 (50–105)	83 (65–111)	72 (50–103)	80 (46–188)	0.426
ALT (IU per l)	41 (35–61)	57 (40–76)	48 (40–68)	44 (36–86)	0.550
Platelet ( × 10^4^ mm^−3^)	8.8 (5.7–11.6)	7 (5.1–9.7)	5.7 (4.7–7.2)	9.4 (6.1–13.7)	0.004
Prothrombin time (%)	64.2 (50.0–68.9)	61 (55.6–66.3)	54.4 (49.4–62.0)	54.1 (45.0–63.0)	0.012
Creatinine (mg per dl)	0.81 (0.70–0.96)	0.76 (0.66–1.10)	0.77 (0.60–0.90)	0.78 (0.61–1.03)	0.593
Ascites (present)	13 (48.2%)	8 (42.1%)	5 (21.7%)	60 (68.2%)	<0.001
Encephalopathy (present)	12 (44.5%)	9 (43.4%)	8 (34.8%)	40 (45.5%)	0.811
Tumour number (single)	9 (33.3%)	7 (36.8%)	17 (73.9%)	28 (31.8%)	0.002
Tumour size (mm)	38 (23–53)	32 (26–65)	20 (17–29)	51.5 (30–100)	<0.001
AFP (ng per ml)	32 (13–1250)	81 (13–706)	21 (9–106)	188 (26–8660)	0.001
Portal invasion (present)	7 (25.9%)	6 (31.6%)	0 (0%)	49 (55.7%)	<0.001
Child-Pugh score (10/11/12∼)	15/11/1	11/6/2	13/7/3	26/23/39	<0.001

Abbreviations: AFP=alpha-fetoprotein; ALT=alanine aminotransferase; AST=aspartate aminotransferase; HBsAg=hepatitis B virus surface-antigen; HCVAb=hepatitis C virus-antibody.

All variables are shown in median (interquartile range) unless otherwise noted.

**Table 2:** Univariate analysis of the prognostic factors of HCC patients with Child C cirrhosis

	**RR**	**95% CI**	***P*-value**
Sex (male)	1.16	0.78–1.78	0.457
Age (per 10years)	1.11	0.91–1.36	0.264
HCVAb (positive)	0.96	0.65–1.44	0.869
HBsAg (positive)	1.21	0.75–1.88	0.404
Total bilirubin (>3 mg dl^−1^)	2.14	1.48–3.09	<0.001
Albumin (>3 g dl^−1^)	1.46	0.94–2.21	0.090
AST (>40 IU l^−1^)	1.04	0.66–1.72	0.843
ALT (>40 IU l^−1^)	1.31	0.90–1.92	0.148
Platelets (>8 × 10^4^ mm^−3^)	2.23	1.52–3.26	<0.001
Prothrombin time (>50%)	1.24	0.84–1.86	0.275
Creatinine (>1 mg dl^−1^)	1.16	0.74–1.76	0.487
Ascites (uncontrollable)	2.46	1.67–3.66	<0.001
Encephalopathy (present)	0.98	0.67–1.41	0.926
Tumour number (multiple)	2.21	1.49–3.31	<0.001
Tumour size (>30 mm)	3.81	2.54–5.83	<0.001
AFP (>400 ng ml^−1^)	2.49	1.69–3.63	<0.001
Portal invasion (present)	3.90	2.64–5.76	<0.001
TACE	0.56	0.33–0.91	0.019
Local ablation	0.42	0.24–0.70	<0.001
TAI	0.97	0.54–1.61	0.915

Abbreviations: TACE=transcatheter arterial chemoembolisation; TAI=transcatheter arterial chemoinfusion. Other abbreviations are as listed in Table 1.

**Table 3:** Multivariate analysis of the prognostic factors of HCC patients with Child C cirrhosis

	**RR**	**95% CI**	***P*-value**
Total bilirubin (>3 mg dl^−1^)	2.94	1.90–4.58	<0.001
Platelets (>8 × 10^4^ mm^−3^)	1.77	1.11–2.84	0.016
Ascites (uncontrollable)	1.80	1.14–2.87	0.010
Tumour number (multiple)	1.67	1.06–2.66	0.025
Tumour size (>30 mm)	3.00	1.74–5.24	<0.001
AFP (>400 ng ml^−1^)	1.68	1.05–2.67	0.029
Portal invasion (present)	1.77	1.09–2.85	0.019
TACE	0.50	0.27–0.89	0.019
Local ablation	1.02	0.51–1.96	0.944
TAI	0.64	0.33–1.16	0.152

Abbreviations are as listed in Tables 1 and 2.

## References

[bib1] Adams PC, Arthur MJ, Boyer TD, DeLeve LD, Di Bisceglie AM, Hall M, Levin TR, Provenzale D, Seeff L (2004) Screening in liver disease: report of an AASLD clinical workshop. Hepatology 39: 1204–1212 15122748 10.1002/hep.20169

[bib2] Bolondi L (2003) Screening for hepatocellular carcinoma in cirrhosis. J Hepatol 39: 1076–1084 14642630 10.1016/s0168-8278(03)00349-0

[bib3] Bruix J, Llovet JM (2002) Prognostic prediction and treatment strategy in hepatocellular carcinoma. Hepatology 35: 519–524 11870363 10.1053/jhep.2002.32089

[bib4] Bruix J, Sherman M, Llovet JM, Beaugrand M, Lencioni R, Burroughs AK, Christensen E, Pagliaro L, Colombo M, Rodes J (2001) Clinical management of hepatocellular carcinoma. Conclusions of the Barcelona-2000 EASL conference. European Association for the Study of the Liver. J Hepatol 35: 421–430 11592607 10.1016/s0168-8278(01)00130-1

[bib5] Camma C, Schepis F, Orlando A, Albanese M, Shahied L, Trevisani F, Andreone P, Craxi A, Cottone M (2002) Transarterial chemoembolization for unresectable hepatocellular carcinoma: meta-analysis of randomized controlled trials. Radiology 224: 47–54 12091661 10.1148/radiol.2241011262

[bib6] Hiraoka A, Michitaka K, Kumagi T, Kurose K, Uehara T, Hirooka M, Yamashita Y, Kubo Y, Miyaoka H, Iuchi H, Okada S, Ohmoto M, Yamamoto K, Horiike N, Onji M (2005) Efficacy of lamivudine therapy for decompensated liver cirrhosis due to hepatitis B virus with or without hepatocellular carcinoma. Oncol Rep 13: 1159–1163 15870937

[bib7] Honda H, Ochiai K, Adachi E, Yasumori K, Hayashi T, Kawashima A, Fukuya T, Gibo M, Matsumata T, Tsuneyoshi M, Masuda K (1993) Hepatocellular carcinoma: correlation of CT, angiographic, and histopathologic findings. Radiology 189: 857–862 8234716 10.1148/radiology.189.3.8234716

[bib8] Ikai I, Arii S, Okazaki M, Okita K, Omata M, Kojiro M, Takayasu K, Nakanuma Y, Makuuchi M, Matsuyama Y, Monden M, Kudo M (2007) Report of the 17th Nationwide Follow-up Survey of Primary Liver Cancer in Japan. Hepatol Res 37: 676–691 17617112 10.1111/j.1872-034X.2007.00119.x

[bib9] Kim YK, Kim CS, Chung GH, Han YM, Lee SY, Jin GY, Lee JM (2006a) Radiofrequency ablation of hepatocellular carcinoma in patients with decompensated cirrhosis: evaluation of therapeutic efficacy and safety. AJR Am J Roentgenol 186: S261–S268 16632686 10.2214/AJR.04.1266

[bib10] Kim YS, Rhim H, Cho OK, Koh BH, Kim Y (2006b) Intrahepatic recurrence after percutaneous radiofrequency ablation of hepatocellular carcinoma: analysis of the pattern and risk factors. Eur J Radiol 59: 432–441 16690240 10.1016/j.ejrad.2006.03.007

[bib11] Lin SM, Lin CJ, Lin CC, Hsu CW, Chen YC (2005) Randomised controlled trial comparing percutaneous radiofrequency thermal ablation, percutaneous ethanol injection, and percutaneous acetic acid injection to treat hepatocellular carcinoma of 3 cm or less. Gut 54: 1151–1156 16009687 10.1136/gut.2004.045203 PMC1774888

[bib12] Llovet JM (2005) Updated treatment approach to hepatocellular carcinoma. J Gastroenterol 40: 225–235 15830281 10.1007/s00535-005-1566-3

[bib13] Llovet JM, Burroughs A, Bruix J (2003) Hepatocellular carcinoma. Lancet 362: 1907–1917 14667750 10.1016/S0140-6736(03)14964-1

[bib14] Makuuchi M, Kokudo N (2006) Clinical practice guidelines for hepatocellular carcinoma: the first evidence based guidelines from Japan. World J Gastroenterol 12: 828–829 16521207 10.3748/wjg.v12.i5.828 PMC4066144

[bib15] Nagasue N, Kohno H, Tachibana M, Yamanoi A, Ohmori H, El-Assal ON (1999) Prognostic factors after hepatic resection for hepatocellular carcinoma associated with Child-Turcotte class B and C cirrhosis. Ann Surg 229: 84–90 9923804 10.1097/00000658-199901000-00011 PMC1191612

[bib16] Parkin DM (2001) Global cancer statistics in the year 2000. Lancet Oncol 2: 533–543 11905707 10.1016/S1470-2045(01)00486-7

[bib17] Parsons LS, Ovation Research Group (2001) Reducing bias in a propensity score matched-pair sample using greedy matching techniques. In: Proceedings of the Twenty-Sixth Annual SAS Users Group International Conference. pp 214–226. Cary: SAS Institute Inc.

[bib18] Pugh RN, Murray-Lyon IM, Dawson JL, Pietroni MC, Williams R (1973) Transection of the oesophagus for bleeding oesophageal varices. Br J Surg 60: 646–649 4541913 10.1002/bjs.1800600817

[bib19] Sala M, Forner A, Varela M, Bruix J (2005) Prognostic prediction in patients with hepatocellular carcinoma. Semin Liver Dis 25: 171–180 15918146 10.1055/s-2005-871197

[bib20] Takamura M, Ichida T, Ohkoshi S, Tsubata S, Osaki A, Aoyagi T, Nomoto M, Uehara K, Terada H, Aoyagi Y (2007) Decompensated lamivudine-resistant hepatitis B virus-related cirrhosis treated successfully with adefovir dipivoxil allowing surgery for hepatocellular carcinoma. Intern Med 46: 367–371 17409599 10.2169/internalmedicine.46.6079

[bib21] Tateishi R, Shiina S, Yoshida H, Teratani T, Obi S, Yamashiki N, Akamatsu M, Kawabe T, Omata M (2006) Prediction of recurrence of hepatocellular carcinoma after curative ablation using three tumor markers. Hepatology 44: 1518–1527 17133456 10.1002/hep.21408

[bib22] Toyoda H, Kumada T, Kiriyama S, Sone Y, Tanikawa M, Hisanaga Y, Hayashi K, Honda T, Kuzuya T, Nonogaki K, Shimizu J (2005) Impact of tumor factors on the prognosis of patients with advanced cirrhosis (Child-Pugh class C) and hepatocellular carcinoma. J Gastroenterol Hepatol 20: 963–965 15946154 10.1111/j.1440-1746.2005.03841.x

[bib23] Trevisani F, Cantarini MC, Labate AM, De Notariis S, Rapaccini G, Farinati F, Del Poggio P, Di Nolfo MA, Benvegnu L, Zoli M, Borzio F, Bernardi M (2004) Surveillance for hepatocellular carcinoma in elderly Italian patients with cirrhosis: effects on cancer staging and patient survival. Am J Gastroenterol 99: 1470–1476 15307862 10.1111/j.1572-0241.2004.30137.x

[bib24] Ueno S, Tanabe G, Nuruki K, Oketani M, Komorizono Y, Hokotate H, Fukukura Y, Baba Y, Imamura Y, Aikou T (2002) Prognosis of hepatocellular carcinoma associated with Child class B and C cirrhosis in relation to treatment: a multivariate analysis of 411 patients at a single center. J Hepatobiliary Pancreat Surg 9: 469–477 12483269 10.1007/s005340200058

[bib25] United Network for Organ Sharing (2006) Annual report 2006. http://www.unos.org/data

[bib26] Wong LL, Limm WM, Severino R, Wong LM (2000) Improved survival with screening for hepatocellular carcinoma. Liver Transpl 6: 320–325 10827233 10.1053/lv.2000.4875

